# Emerging Concepts and Challenges in Rheumatoid Arthritis Gene Therapy

**DOI:** 10.3390/biomedicines8010009

**Published:** 2020-01-09

**Authors:** Andrei A. Deviatkin, Yulia A. Vakulenko, Ludmila V. Akhmadishina, Vadim V. Tarasov, Marina I. Beloukhova, Andrey A. Zamyatnin Jr., Alexander N. Lukashev

**Affiliations:** 1 Institute of Molecular Medicine, Sechenov First Moscow State Medical University, 119048 Moscow, Russia; beloukhovamarina@gmail.com (M.I.B.); zamyat@belozersky.msu.ru (A.A.Z.J.); alexander_lukashev@hotmail.com (A.N.L.); 2 Martsinovsky Institute of Medical Parasitology, Tropical and Vector Borne Diseases, Sechenov First Moscow State Medical University, 119435 Moscow, Russia; vjulia94@gmail.com (Y.A.V.); akhludmilav@gmail.com (L.V.A.); 3 Faculty of Biology, Lomonosov Moscow State University, 119234 Moscow, Russia; 4 Department of Pharmacology and Pharmacy, Sechenov First Moscow State Medical University, 119991 Moscow, Russia; tarasov-v-v@mail.ru; 5 Belozersky Institute of Physico-Chemical Biology, Lomonosov Moscow State University, 119992 Moscow, Russia

**Keywords:** rheumatoid arthritis, gene therapy, AAV, miRNA, siRNA, lncRNA, circRNA, monoclonal antibodies, gene delivery, CIA, clinical trials

## Abstract

Rheumatoid arthritis (RA) is a systemic inflammatory joint disease affecting about 1% of the population worldwide. Current treatment approaches do not ensure a cure for every patient. Moreover, classical regimens are based on nontargeted systemic immune suppression and have significant side effects. Biological treatment has advanced considerably but efficacy and specificity issues remain. Gene therapy is one of the potential future directions for RA therapy, which is rapidly developing. Several gene therapy trials done so far have been of moderate success, but experimental and genetics studies have yielded novel targets. As a result, the arsenal of gene therapy tools keeps growing. Currently, both viral and nonviral delivery systems are used for RA therapy. Herein, we review recent approaches for RA gene therapy.

## 1. Introduction

Gene therapy is an experimental technique that involves the administration of specific DNA or RNA to modify gene expression in order to treat or prevent diseases. The concept of gene therapy is almost 40 years old, and the first meaningful studies were done around 30 years ago [1]. Despite several successful clinical trials, gene therapy remains a theoretical possibility due to a high cost of development and manufacturing as well as safety concerns. However, it gives hope in those cases where conventional drugs fail to provide the desired therapeutic effect. Rheumatoid arthritis (RA) is an autoimmune disease that affects about 1% of the world’s population [2,3]. The main problem in RA treatment is that the exact causes of the disease are unknown [4]. Obviously, a genetic predisposition is among the major factors, because the prevalence varies in different populations. For example, the native tribes of North America have a higher RA prevalence (up to 7%) as compared to African and Asian populations (0.2–0.4%) [5]. The incidence of RA increases after 25 years of age and reaches a plateau by 55 years of age. Females have a greater incidence than males, with a 2–3:1 ratio, which can be explained by a possible role of hormones in the pathogenesis of this autoimmune disease [6].

Although the etiology of RA remains unknown, it is undoubtedly an autoinflammatory disease. Early RA treatment may include glucocorticoids that reduce inflammation by upregulation of anti-inflammatory signaling and downregulation of proinflammatory signaling. Early RA treatment may include glucocorticoids that reduce inflammation by upregulation of anti-inflammatory signaling and downregulation of proinflammatory signaling. However, substantially adverse effects have limited their use [7]. Nonsteroidal anti-inflammatory drugs (NSAIDs) can be used for symptomatic treatment of RA but have limited efficacy and cause significant side effects, especially upon prolonged administration [8]. The next class of remedies, disease-modifying antirheumatic drugs (DMARDs), are heterogeneous agents grouped by use and convention. They reduce joint swelling and pain, decrease acute-phase biochemical markers in the serum, limit progressive joint damage, and improve function [7]. Paradoxically, the target of the dominant DMARD, the immunosuppressant methotrexate (MTX), is unknown [9]. However, to date, MTX is the first-line treatment for RA. MTX produces clinical remission, which is accessed by the European League Against Rheumatism (EULAR) score, in 25–50% of patients [10]. The next major development in RA therapy was the biological DMARDs (bDMARDs), a group of functionally different classes of drugs, primarily monoclonal antibodies (mABs).

According to the Kyoto Encyclopedia of Genes and Genomes (KEGG), there are dozens of proteins involved in the pathogenesis of RA [11]. Binding of these proteins by specific mABs may block the progression of RA. In the early 2000s, this technology emerged as a promising breakthrough. Tumor necrosis factor inhibitors (TNFis) are the most frequently prescribed class of bDMARDs for the treatment of patients with RA who are nonresponsive to MTX [10]. Moderate and good EULAR responses at 6 months were achieved in 69% and 40% of the patients receiving the first TNFi, respectively [12]. Further improvement is possible by using combinations of drugs; however, the absence of efficacy predictors limits personalized optimization of treatment.

**Figure 1:**
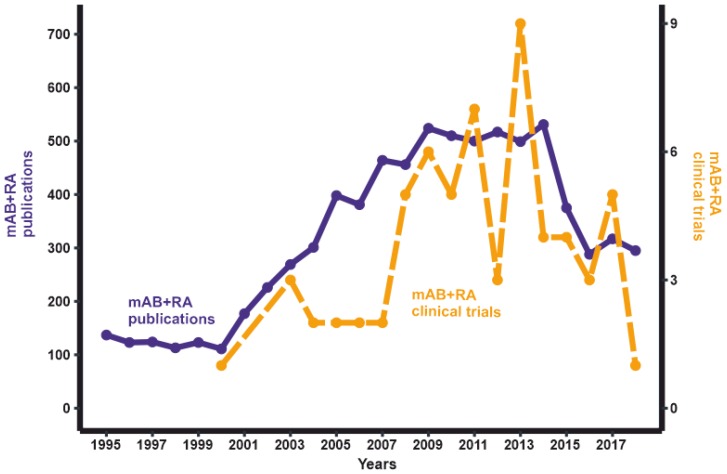
The dynamics of the publications/clinical trials number with keywords “monoclonal antibody” AND “rheumatoid arthritis” as of October 2019.

As of October 2019, clinicaltrials.gov has information on 70 completed clinical trials of RA treatment using mABs [13]. The completion date is known for 65 of these clinical trials. The number of successfully completed RA therapy clinical trials peaked in 2013 (Figure 1). Noteworthy, the average duration of clinical trials (the difference between “Start Date” and “Completion Date”) was 2.7 years. Hence, the number of novel mAB drug trials has been decreasing over time. The same trend is observed for the number of publications in PubMed found with the query “monoclonal AND rheumatoid arthritis” (Figure 1). This may be because mABs to the most promising targets for RA treatment have already been tested, and further options in this direction are almost exhausted. Besides bDMARDs, a new category of drugs, Janus kinase inhibitors (jakinibs), can be used in the treatment of RA [14]. These small molecules inhibit the Janus kinase enzymes, which are components of the Janus kinase-signal transducer and activator of transcription (JAK-STAT) signaling pathway [15]. The JAK-STAT pathway regulates the level of transcription and expression for more than 50 cytokines and growth factors in response to external signals. Blocking the components of this pathway can modulate immune response and has potentional in treating RA [16].

Development of gene therapy technologies has the potential to open a new dimension in RA therapy. However, the potential of gene therapy in RA remains unclear. The purpose of the current review is to cover the current state of this problem and future directions for RA gene therapy development.

## 2. Targets for RA Gene Therapy

### 2.1. Anti- and Proinflammatory Cytokines

The overproduction of inflammatory cytokines by fibroblast-like synoviocytes (FLSs) and activated immune cells is believed to play a crucial role in the development and progression of RA [17]. The inhibition of proinflammatory cytokines and/or overexpression of anti-inflammatory cytokines is one of the mainstream strategies in RA treatment, which has been explored since the early 2000s [18].

The key proinflammatory cytokines involved in the pathogenesis of RA are tumor necrosis factor alpha (TNF-α), interleukin 1 (IL-1), and IL-6. Reducing the activity of these cytokines via administration of mABs or soluble receptors decreased the signs and symptoms of RA. Several mABs against TNF-α (infliximab, adalimumab, certolizumab pegol, etanercept, and golimumab), IL-1 (anakinra), and the IL-6 receptor (IL6-R) (tocilizumab) are currently approved by the Food and Drug Administration (FDA). Anti-TNF therapy showed good efficacy in combination with MTX, and this treatment is currently suggested as the first-line RA therapy. The IL6-R inhibitor tocilizumab appears to be more effective as monotherapy [18]. Therapy with biologicals retards the progression of RA and preserves the functional status of involved joints. However, a significant fraction of patients with RA (10–20%) do not respond to any of the existing therapies [19]. Thus, attempts to find new targets for treating RA are ongoing.

Several studies have been aimed at inhibiting other proinflammatory cytokines (Table 1). The suppression of IL-18 [20,21], IL-33 [22], and IL-19 [23] showed a significant therapeutic effect in collagen-induced arthritis (CIA) animal models in mice and rats, but no clinical trials have been performed to date. The phase II clinical trials of mABs against IL-15 [24], IL-20 [25], and IL-22 [26] showed disappointing results in patients with RA. The phase III study of secukinumab, an IL-17A inhibitor, demonstrated efficacy in patients that did not respond to anti-TNF therapy. Its efficacy did not exceed that of abatacept, which is already approved for use in patients with an inadequate response to TNFis. Together with the results of phase II trials of the other IL-17 inhibitors, this might suggest that the role of IL-17 in the pathogenesis of RA is more modest than has been suggested earlier [27]. Thus, targeting of several promising ILs did not show the desired effect, stressing the need for novel directions in RA therapy development.

The administration of anti-inflammatory cytokines might be another strategy for RA treatment. Gene therapy with IL-4 and IL-10 in CIA animal models demonstrated joint protection and reversed cartilage degradation, despite high inflammation levels [28,29]. However, clinical trials of these cytokines administered alone showed low efficacy. The synergetic activity of IL-4 and IL-10 suggested a novel approach—using a fusion protein of IL-4 and IL-10 (IL4-10 FP) to treat RA (Table 1) [30]. Glycosylated IL4-10 FP was shown to decrease the severity of proteoglycan-induced arthritis (PGIA) in mice [30]. While further investigations are required to prove its potential as a drug, the use of fusion proteins of two inflammatory or regulatory cytokines appears to be a promising approach and a potential direction for RA gene therapy development.

It is important that, in some cases, overexpression of anti-inflammatory cytokines may lead to unexpected results. Administration of the immunosuppressive cytokine IL-35 significantly exacerbated the course of RA in animal models [31]. This result suggests more complex mechanisms of the pathogenesis of RA than were previously thought.

### 2.2. Matrix Degradation Enzymes

Joint destruction in RA is mediated by the increased activity of matrix-degrading proteinases, proteolytic enzymes that degrade connective tissue. Two classes of matrix-degrading proteinases are involved in the pathogenesis of RA: matrix metalloproteinases (MMPs) and disintegrin-metalloproteinases with thrombospondin motifs (ADAMTSs). They are produced by different cell types in synovial tissue, notably by macrophages and FLSs activated by inflammatory cytokines, growth factors, and intercellular interactions [32]. While some MMPs are constitutively expressed in cartilage and upregulated in RA (MMP-1, MMP-2, MMP-13, and MMP-14) [33], the others (MMP-3, MMP-8, and MMP-9) appear to be specific markers of pathologic conditions [34,35]. Specifically, the levels of MMP-1 and MMP-3 correlate with disease severity [36]. MMPs are functionally involved in the pathogenesis of RA. Several attempts to inhibit specific MMPs in order to treat RA were made in the 2000s. However, the use of synthetic inhibitors of MMPs in RA treatment failed to show efficacy in clinical trials. These inhibitors caused various adverse effects due to their lack of selectivity [37]. Nevertheless, the advances in structural biology and drug design yielded novel molecules with desired selectivity, which might be promising therapeutic agents [38].

Several MMPs are of particular interest in the pathogenesis of RA. The inhibition of MMP-9 in RA-FLSs, which is highly expressed in cartilage with RA, promoted the survival, invasion, and the release of proinflammatory cytokines by FLSs (Table 1) [39]. The phase Ib clinical trials of the mAB against MMP-9, andecaliximab, proved short-term safety [40]. MMP-14 is the most prominent among the membrane-bound MMPs. It is also referred to as membrane type 1 MMP (MT1-MMP). MMP-14 promotes the invasion of RA-FLSs via cartilage collagen degradation. In the mouse CIA model, the inhibition of MT1-MMP by mABs resulted in the reduction of cartilage degradation [41]. 

It is unlikely that MMPs may be the sole target of RA therapy. However, targeting them may increase the efficacy of gene therapy drugs, especially in the context of intra-articular delivery. Importantly, in some cases, they can be added to multitarget gene therapy as secondary targets, which would come at no additional cost in the case of large-capacity delivery vehicles.

### 2.3. Hormonal Regulation Proteins

Although the etiology of RA remains unclear, some pathogenesis patterns are well-known. The disease is more prevalent in women, suggesting the possible significance of sex hormones, but the mechanism of the relationship between sex hormones and RA remains obscure. Recently, it was demonstrated that estrogen receptor deletion in T cells reduced their activation and proliferation rates. One study suggested that using estrogen receptor targeted immunotherapies could treat autoimmune disorders [42]. 

Circadian rhythms could be also involved in the development of RA. Seasonality significantly affects the prevalence of RA [43,44], and women living in higher latitudes may be at greater risk for RA [45]. Recently, melatonin has been proposed to be a link between circadian rhythms and RA [46]. This hormone has the lowest level in spring, which strongly correlates with the highest exacerbation rates of another autoimmune disease, multiple sclerosis [44]. Melatonin, in addition to its other functions, may inhibit the estrogen receptor [47]. However, we could not find any reports of melatonin-based RA therapies.

### 2.4. Noncoding RNA Molecules

#### 2.4.1. MicroRNAs

MicroRNAs (miRNAs) are small noncoding RNA molecules about 22–23 nucleotides in length. They play an essential role in the regulation of gene expression in animals and plants through pairing with the mRNAs of protein-coding genes and inhibiting their translation [48]. MiRNAs are involved in regulation of the inflammatory response in immune and non-immune cells [49]. Expression of some miRNAs, such as miR-155 (miRNA-155) and miR-146a, was found to be upregulated in different cell types and tissues in patients with RA [50,51]. However, for many miRNAs, their exact mechanistic role in the pathogenesis of RA is unknown.

Recent studies have reported several miRNAs that modulate the function of FLSs (Table 1). Specifically, the effects of some miRNAs on FLSs in RA have been reviewed in [52]. MiRNAs may regulate the production of cytokines, chemokines, and MMPs. The basal expression of miR-203 was higher in RA-FLSs than in FLSs from healthy donors, and its overexpression in RA-FLSs led to the significantly increased levels of IL-6 and MMP-1 [53]. The expression of miR-17~92 cluster in RA-FLSs was upregulated with TNF-α in nuclear factor kappa-light-chain-enhancer of activated B cells (NF-κB)-dependent manner. The transfection of RA-FLSs with the precursor of miRNA-18a, the member of miR-17~92 cluster, increased levels of MMP-1 and pro-inflammatory cytokines IL-6 and IL-8 [54]. The levels of miR-17 expression were lowered in serum, FLSs, and synovial tissues of patients with RA as well as in the serum and joints of adjuvant-induced arthritis (AIA) rats. MiR-17 targeted the ubiquitin proteasome system (UPS) acting as a negative regulator of TNF-α signaling. The transfection of RA-FLSs with miR-17 precursor inhibited TNF-α-induced IL-6, IL-8, MMP-1, and MMP-13 production in RA-FLSs. This indicates the possible protective role of miR-17 in cartilage destruction [55]. MiR-19 directly targeted Toll-like receptor 2 (TLR2) in RA-FLS. The overexpression of miR-19 decreased TLR2 expression and downregulated IL-6 and MMP-3 in lipopolysaccharides(LPS)-stimulated RA-FLSs [56]. MiR-20 regulated the secretion of pro-inflammatory cytokines. This miRNA targeted apoptosis signal-regulating kinase (ASK) 1, a key component of the toll-like receptors 4 pathway, which caused downregulation of IL-6 and CXCL10 in LPS activated RA-FLSs [57].

Several miRNAs have been demonstrated to modulate cell invasion and apoptosis of RA-FLSs (Table 1). Downregulation of miR-135a inhibits proliferation, migration, and invasion and promotes apoptosis of RA-FLSs [58]. Inhibition of miRNA-21 in RA-FLSs in rats with CIA led to a significant decrease in the proliferation of RA-FLSs. At the same time, the proliferation rate was increased when this miRNA was overexpressed in normal FLSs [59]. miR-124a was shown to suppress proliferation of FLSs through inhibiting the expression of MMP-3/13 and IL-1β [60]. The expression of miR-27a was significantly decreased in serum, synovial tissues, and FLSs of patients with RA as compared with healthy controls [61]. It targets the proinflammatory mediator follistatin-like protein 1 (FSTL1), and overexpression of miR-27a downregulates the expression of MMPs (MMP2, MMP9, and MMP13) and Rho family proteins (Rac1, Cdc42, and RhoA).

#### 2.4.2. Long Noncoding RNAs

Long noncoding RNAs (lncRNAs) are a large class of regulatory RNAs with lengths exceeding 200 base pairs. The mechanisms of lncRNA regulation are based on their ability to form complex secondary structures that interact with proteins, DNA, and other RNAs. lncRNAs are involved in the regulation of gene expression, cell proliferation, differentiation, and immune response [62]. Recent studies have highlighted the essential role of lncRNAs in immune responses and suggested that lncRNAs are involved in the development of autoimmune diseases [63,64,65,66].

lncRNAs modulate different cytokines (e.g., IL-6 or TNF-α) in immune cells during the pathogenesis of RA [67]. The homeobox transcript antisense RNA (HOTAIR) lncRNA has notably higher expression levels in peripheral blood mononuclear cells (PBMCs) and extracellular serum exosomes in patients with RA [68]. The analysis of HOTAIR-mediated pathways suggested that axin, the scaffold protein in the Wnt signaling pathway, is a possible therapeutic intervention point [69].

The therapeutic effects of several other lncRNAs are listed in Table 1. Gastric adenocarcinoma predictive long intergenic noncoding RNA (GAPLINC) is overexpressed in FLSs from patients with RA [70]. Lowly expressed in rheumatoid fibroblast-like synoviocytes (LEFRS) lncRNA is a negative regulator of the proliferation and migration of FLSs [71]. On the contrary, zinc finger NFX1-type containing 1 antisense RNA 1 (ZFAS1) is overexpressed in FLSs and positively regulates the invasion and migration of FLSs. Nuclear enriched abundant transcript 1 (NEAT1) is overexpressed in the PBMCs of patients with clinical RA. The knockdown of NEAT1 lncRNA restrained immune cell differentiation and thus relieved inflammation in CIA mice [72]. A recent study revealed that ENST00000483588 was upregulated, whereas three other lncRNAs (ENST00000438399, uc004afb.1, and ENST00000452247) were downregulated in RA-FLSs [73].

The latest database of lncRNAs described in the literature was updated in 2015 [74]. It contains 294 functionally annotated lncRNAs. At the same time, tens of thousands of lncRNAs have been described to date [75]. Thus, the partial function of only a small percentage of lncRNAs is clear. Several already well characterized lncRNAs and other functionally yet unannotated lncRNAs are potential novel targets for RA gene therapy.

#### 2.4.3. Circular RNAs

**Figure 2:**
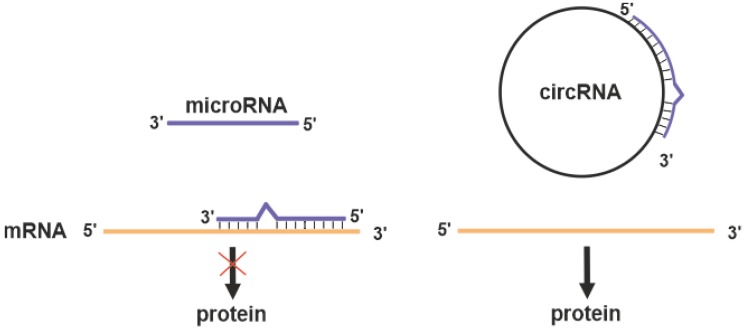
MicroRNA inhibits the translation of target mRNA (red cross on the black arrow). Circular RNAs (circRNA) may act as competitive inhibitor of microRNA preserving target protein expression.

Circular RNAs (circRNAs) are a novel class of transcribed RNAs found in Archaea [76] and a variety of eukaryotic organisms [77,78]. High-throughput RNA-sequencing has shown that at least 20% of currently active genes express circRNAs [79]. Usually, circRNAs are formed through “back-splicing” of the precursor messenger RNAs (pre-mRNAs), in which a downstream splice donor is joined to an upstream splice acceptor via a 3’→5’ phosphodiester bond [80,81,82]. CircRNAs contain several regions complementary to target miRNAs referred to as miRNA response elements. This enables circRNAs to function as microRNA sponges (Figure 2) and regulate many biological processes, including DNA methylation, immune responses, and inflammatory responses [78].

CircRNAs have been shown to play an essential role in various physiological and pathological processes, such as cancer, cardiovascular diseases, neuronal diseases, and autoimmune diseases. A number of circRNAs are differentially expressed in the synovial tissues of patients with RA [83,84]. The molecular mechanisms underlying regulation by circRNAs require further investigation. Nevertheless, the high-throughput sequencing studies of circRNA expression [85] are of particular interest, as the potential miRNA targets of circRNAs can be inferred using bio informatic approaches [86].

## 3. RA Gene Therapy Delivery

### 3.1. Local or Systemic Delivery

In gene therapy, effector delivery may be local or systemic [90]. For local delivery, the drug is injected into the compartment requiring therapy or in the immediate vicinity. The clinical significance of this approach was demonstrated during the treatment of severe heart failure [91] and inherited blindness [92]. Systemic delivery involves the introduction of an effector into the circulatory or lymphatic system. For example, a systemically administered gene therapy targeting liver was successfully used to treat severe hemophilia B [93].

RA is a systemic disease that affects all joints, albeit to different degrees. Its severity is measured by the Disease Activity Score (DAS) [94], which is a criterion based on counting tender and swollen joints. Initially, 44 joints were evaluated. Later, it was demonstrated that evaluating 28 joints was as valid and reliable as more comprehensive joint counts [95]. Local delivery would imply injecting medicine into every swollen joint, which may be tedious. Thus, systemic delivery is preferable for RA treatment [96]. At the same time, systemic delivery may have systemic side effects. Combinations of these approaches have been suggested [97], and they will be discussed later.

### 3.2. Ex Vivo or In Vivo

Genetic modifications of human cells can be done either by an ex vivo approach, when cultured autogenic cells are modified and subsequently administered to the patient or an in vivo approach, when changes are made directly in the human organism [98]. Both methods are possible in RA treatment and have been used in dozens of studies (reviewed in [99]). A common drawback observed in several ex vivo studies is that modified cells are cleared in 2 weeks after intra-articular injection. Thus, in vivo delivery is technically preferable for RA treatment. On the other hand, ex vivo treatment allows circumventing serious regulatory issues associated with gene therapy and may be chosen for this reason.

### 3.3. RNA Therapeutics

#### 3.3.1. RNA Interference

Currently, several therapeutic strategies are employed to modulate RNA function in cells. RNA interference (RNAi) is a process in which miRNA and small interfering RNA (siRNA) molecules interact with complementary RNA molecules, causing their degradation. Dozens of known proteins involved in the pathogenesis of RA either contribute to the development of the inflammatory process or suppress it. RNAi therapy is mainly based on interfering with inflammation-inducing proteins. RNAi delivery systems targeting TNF, NF-kB, and complement system have been extensively explored in animal models of RA [100].

Upon its discovery, RNA silencing technology appeared as a potential game-changer in therapeutics development. However, 20 years of development resulted in just one product suitable for clinical application. Patisiran, an investigational RNAi therapeutic agent that specifically inhibits hepatic synthesis of transthyretin, was FDA-approved in 2018 [101]. Thus, the clinical prospects of RNAi technology in RA remain obscure.

Another promising nucleic acid therapy, antisense oligonucleotide (ASO), may potentially be used for RA treatment. ASOs are defined as chemically synthesized (desoxi)ribooligonucleotides, generally 12–30 nucleotides in length, that are designed to bind to RNA by Watson–Crick base pairing [102]. Following binding to the target RNA, ASO modulates RNA function by promoting its degradation, polyadenylation inhibition, splicing modulation, or translation inhibition. Recently, the first ASO-based drug (inotersen) was approved for the treatment of hereditary transthyretin (hATTR) amyloidosis [103]. Both inotersen or patisiran target the same disease—hATTR amyloidosis polyneuropathy, for which the only first-line treatment available previously was orthotopic liver transplantation [104]. These agents substantially reduce transthyretin (TTR) protein levels by degrading TTR mRNA, specifically degradation of mRNA via nuclear RNaseH1 with inotersen or cytoplasmic RNA-induced silencing complex with patisiran [105].

#### 3.3.2. Circular RNA

CircRNAs not only represent the possible targets for RA (see above) but also may be used as effectors targeting miRNA/siRNA [79]. CircRNAs are much more stable than mRNAs and may produce proteins. Thus, exogenous circRNA itself can be used as a vehicle for gene delivery, providing robust and stable protein expression in eukaryotic cells [106]. In the context of RA, information on circRNA is too fragmentary to make far-reaching conclusions but may be one of the hot fields in the near future. It is noteworthy that, in cancer research, miRNAs were initially supposed to be “oncogenic” or “oncosupressors”. Later, a more in-depth investigation made it clear that miRNAs are too versatile to be classified as “good” or “bad” [107]. Similarly, it is inevitable that the novel functions of circRNAs would be discovered.

#### 3.3.3. Nonviral RNA Delivery Vehicles

The major obstacle to the development of RNA therapeutics is the lack of a suitable vehicle for safe and efficient delivery to diverse cell populations after systemic injection. One of the main functions of the lipid bilayer is preventing RNA and macromolecules on the outside from invading the inside of cells [108]. However, recent advances in chemistry promise to hack this evolutionary armor.

In CIA animal models, small peptide carriers were used as delivery vehicles for siRNA. Mice injected with TNF-α siRNA complexed with rabies virus glycoprotein fused to 9 arginine (RVG-9R) peptide demonstrated joint pathology reduction [109]. Another peptide, melittin, is a cationic amphipathic component of bee venom. Melittin or its derivatives may be used for oligonucleotide delivery [110]. Polymerized siRNA (poly-siRNA) forms stable complexes with thiolated glycol chitosan (tGC) polymers through electrostatic interactions and chemical cross-links. This was used for the delivery of siRNA targeted to TNF-α mRNA, producing reduction of the clinical scores and hind paw swelling in mice with CIA [111]. Polyethylenimine (PEI) can deliver siRNA causing silencing of TNF-α. Cytotoxic PEI properties can be significantly reduced by synthesizing its biodegradable derivative. The complex of biodegradable PEI and TNF-α siRNA decreased the severity of arthritis in CIA mice [112]. Numerous other studies have been carried out demonstrating nonviral RNA delivery (reviewed in [100]). Therefore, currently there are several promising RNA delivery approaches, but none of them are clearly superior to others. This may be the beginning of a new field in gene therapy, but further studies are needed to optimize and refine these methods [113].

### 3.4. (Epi)Genome Editing

**Figure 3:**
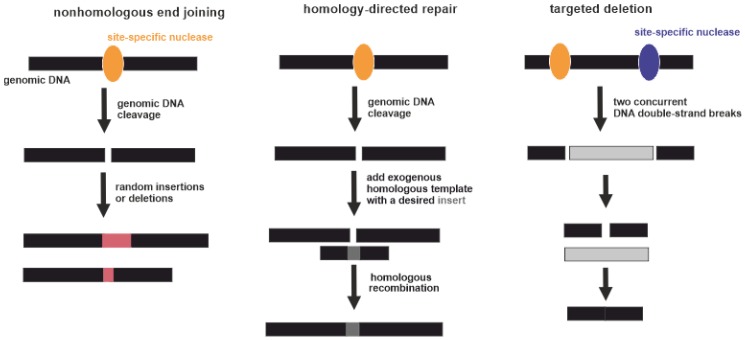
Three types of gene-specific manipulations. Black boxes indicate genomic DNA. Yellow and violet ellipses indicate site-specific nucleases. Pink boxes indicate random insertions or deletions acquired during nonhomologous end joining. Dark grey box indicates a desired insert acquired during homology-directed repair. Light grey box indicates excised part of genomic DNA between two DNA double-strand breaks.

Genome editing techniques make use of certain proteins that can cut DNA at a precise, targeted location. Point mutations in over 3000 genes may be associated with disease development [114]. Potentially this can be corrected by genome editing. Three types of gene-specific manipulations can be conducted (Figure 3). Targeted mutagenesis interrupts gene function by adding random insertions or deletions during nonhomologous end joining. Homology-directed repair is produced by homologous recombination between the original and exogenous gene copies [115]. Additionally, large genomic regions can be deleted by multiple double-strand breaks [116]. Currently, four genome-editing nucleases are commonly used: meganucleases (MNs), zinc-finger nucleases (ZFNs), transcription activator-like effector nucleases (TALENs), and CRISPR-associated nucleases [117]. Regarding RA, the exact gene that should be unequivocally edited or replaced is unknown. Some genome-wide association studies (GWASs) demonstrated a strong association of several SNPs with the severity of RA. However, the pathogenic mechanisms of RA have yet to be fully elucidated [118]. Targeted gene editing RA therapies require a more detailed understanding of its etiology [116].

It is also possible to induce demethylation of methylated cytosine (5mC) at CpG sites and revert histone modifications [119]. At the same time, it is hardly conceivable to identify a sole target for RA treatment, because multiple epigenetic factors acting in distinct cells are collectively involved in the pathogenesis and progression of the disease [4]. To the best of our knowledge, there have been no experimental studies on RA treatment via genome or epigenome editing.

### 3.5. Viral Vectors

The first gene therapy aimed at introducing a functional gene to complement the function of a defective gene in patients with monogenic diseases. For example, in the mid-1990s, the transmembrane regulator of cystic fibrosis was introduced into the respiratory epithelium of patients with cystic fibrosis [120], dystrophin into the muscle cells of patients with Duchenne myodystrophy [121], and low-density lipoprotein receptor into the liver of patients with familial hypercholesterolemia [122]. 

Initially, gene therapy promised hope to cure almost any genetic disease. These expectations were not realized. Clinical trials did not demonstrate an appropriate level of effectiveness. The main obstacle was a lack of specific methods to deliver therapeutic genes to the target tissue, because early viral vectors did not persist in the cells of the host organism. As a result, a sustainable effect could not be achieved [123]. This issue appeared critical for the future of gene therapy, since technologies for identifying genes associated with diseases developed much faster than techniques for safe delivery of nucleic acids into cells [124]. 

One of the first gene therapy delivery vehicles was viruses. These agents have naturally developed a perfect capacity to deliver nucleic acids to the host cells. A variety of viruses with unique properties allows choosing the most appropriate vehicle for each application [125,126]. However, certain viral vectors may cause significant side effects. The most notorious example is the first case of human death reported in 1999 in the course of a promising gene therapy drug testing. That study focused on the treatment of ornithine carbamoyl transferase deficiency using a modified adenovirus as a vector [127]. A combination of unjustified expectations and tragic disappointments resulted in significant retardation of gene therapy development. It was concluded that it is necessary to profoundly understand the characteristics of each particular vector used in gene therapy before its clinical implication [123].

Adenoviruses were proposed for use in gene therapy more than 20 years ago [128]. Adenoviruses are non-enveloped viruses with a double-stranded DNA genome of about 35,000 base pairs. Adenoviruses are attenuated by removing some of the “early” genes to be usable for gene therapy. First-generation adenoviral vectors lacked only the E1B gene. Second-generation vectors were developed by omitting most of the early genes. Gutless adenovirus-based vectors contain only terminal repeats from the adenovirus genome and target sequence, offering the best safety [129]. The large size of the genome made it possible to insert up to 2000 nucleotides into early adenoviral vectors and up to 30,000 nucleotides into the gutless type.

**Figure 4:**
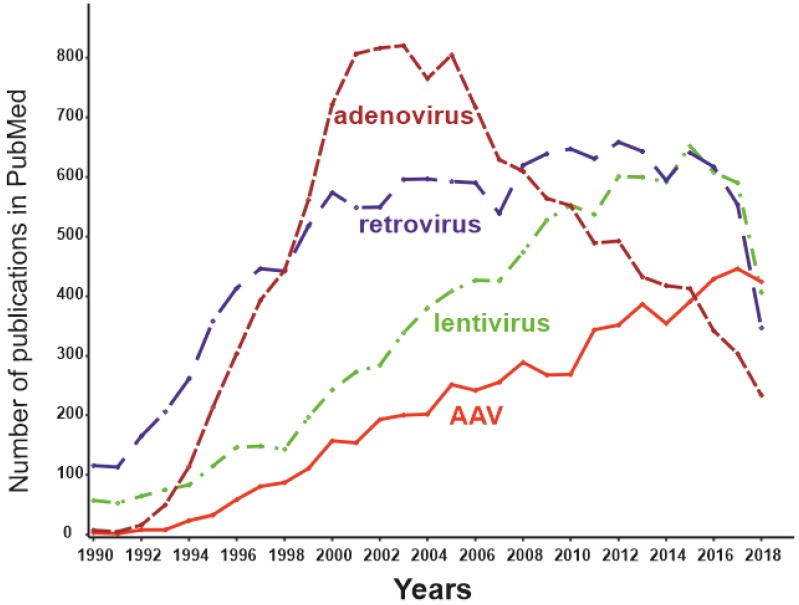
The dynamics of the number of publications with keywords “adeno-associated virus” AND “gene therapy” (red), “lentivirus” AND “gene therapy” (green), “retrovirus” AND “gene therapy” (blue), and “adenovirus” AND “gene therapy” (brown) as of May 2019.

Initially, adenoviral vectors were suggested to be used to treat a wide range of diseases. Later, it turned out even genetically inert virus particles contain motifs that could trigger an extremely potent innate immune response in the case of a systemically administered adenoviral vector [127]. For this reason, adenoviral vectors are not currently the mainstream virus vectors (Figure 4) and are used primarily for applications in which an immune response is desirable and local administration of the effector is possible (e.g., therapy of malignant tumors or vaccine development). Certainly, autoimmune RA is not among the optimal applications for adenoviral delivery systems.

Retroviruses are notorious for their capacity to cause AIDS and a range of malignancies. Besides, they are some of the most commonly used gene therapy vehicles. The first clinical trials of gene therapeutic drugs based on retroviruses were carried out 29 years ago [130]. This was an attempt to insert a “correct” copy of the adenosine deaminase gene into the T-lymphocyte genome of patients with severe combined immunodeficiency mediated by a lack of this enzyme. At that time, retroviruses were the only feasible way to modify the host DNA, because their life cycle includes integration into the host genome. Initially, constructs based on the murine leukemia virus (MuLV) were used as vectors. MuLV inserts the target fragment predominantly at the beginning of random transcription units of the host genome, which can lead to dysregulation of the expression of the oncogene/oncosupressor. This has led to the development of malignancies during retroviral gene therapy [131,132]. Such consequences can be avoided using a specific type of retrovirus: self-inactivating lentivirus [133]. However, the risk of malignancies cannot be fully excluded. Thus, the introduction of lentivirus-based constructs into clinical practice is limited and must include a very careful risk/benefit analysis, which is unlikely to be favorable in the case of RA.

Certain viruses can persist without causing overt disease and thus may make promising gene therapy vectors [134]. Adeno-associated viruses (AAVs) are among such persisting benign viruses. Currently, vectors created on the basis AAVs are recognized as safe and effective, being one of the most promising methods of gene therapy [135]. The number of publications dedicated to AAVs peaked in 2018 (Figure 2), while the use of other virus vectors has been declining. AAVs are small non-enveloped viruses with a dsDNA genome of about 4800 nucleotides. AAVs are not able to replicate independently and require several proteins complemented by adenoviruses, hence the name [124]. Viral ssDNA genome penetrates the nucleus after host cell infection. Most of the viral genomes do not integrate into the genome of the host cell, remaining in the form of episomes. At the same time, approximately 0.1% of the naturally occurring AAV genomes can be integrated into the genome, with integration taking place at a specific location in the 19th human chromosome. Recombinant AAV episomal DNA lacks the ability to integrate into host genomic DNA. Thus, the genotoxic effect inevitable for retroviral constructs has not been observed using AAV vectors [136]. As the AAV genome exists as an epichromosome, it can be removed by the cell, as well as diluted by successive cell division. Thus, the number of viruses in dividing cells is gradually reduced. This limits the use of AAVs in gene therapy to short-term gene delivery and transfection of slowly dividing cells. In the mid-1990s, it was shown that transgenes delivered to rat neural tissue by an AAV vector was stably expressed for at least several months [137]. A decade later Mochizuki et al. demonstrated correction of hyperphenylalaninemia by AAV-mediated gene transfer in mice with phenylketonuria. Interestingly, within 2 weeks of treatment, all animals had no symptoms of the disease, and the therapeutic effect persisted for 40 weeks only in male mice [138]. The main cellular components of the synovium and the most promising targets for RA gene therapy are FLSs and macrophage-like synoviocytes (MLSs) [139]. Due to their epichromosomal nature, AAVs are “diluted” during cell division. The lifespan of MLSs is estimated to be 3–6 months [140], and they can be maintained for several months in vitro [141]. Thus, the key cells involved in the pathogenesis of RA are not fast-dividing cells; therefore, AAV-based technology is generally appropriate for RA gene therapy.

Another natural limitation of AAVs is the relatively low coding capacity of the vector. DNA fragments over 4000 nucleotides in length cannot be efficiently transduced [142]. This can be circumvented by using a dual vector system [143]. Dysferlin-deficient mice were treated systemically by two AAV vectors to provide reconstitution of the full 6.5 kb dysferlin cDNA. The treatment provided long-term gene expression and functional improvement for dysferlinopathy [144]. However, the clinical usability of such systems remains questionable. 

Currently, 13 natural AAV serotypes are known and are termed AAV1–AAV13 [145]. Noteworthy, by definition, a serotype (serological type) is a serologically distinguishable strain of a virus. Strikingly, AAV6, AAV10, and AAV11 in these terms represent one serotype [146]. The serological features of the more than 100 other AAV strains are not currently known; hence, these strains are termed AAV variants. These hundreds of uncharacterized variants potentially can represent novel serotypes. Known AAV serotypes have tropism to different human tissues. For example, in mice, the primary targets for AAV serotypes 1, 2, 5, 6, 7, and 9 are the liver and hindlimbs. AAV8 and 9 transduce tissues more ubiquitously in comparison with other serotypes. A transgene delivered by AAV9 was detected, besides other targets, in tissues protected by additional barriers, such as the testes and brain [147]. According to other authors, AAV1 is targeted to the nervous system and skeletal muscle; AAV2 has broad tropism; AAV5 is targeted to retinal pigmented epithelia and photoreceptors; AAV6 to skeletal muscle and lung; AAV8 to the liver, skeletal muscle, heart, and pancreas; and AAV9 to the liver, skeletal muscle, and lung [124]. An AAV directed evolution technique allows to select from a variety of synthesized variants the one most suitable for a particular application. Moreover, the wild type AAV encoding capsid can be modified to create gene libraries and then select the most appropriate novel variants [135]. For example, a novel recombinant AAV-PHP.B transfers genes after systemic administration throughout the CNS with an efficiency that is at least 40-fold greater than that of the natural viruses [148]. Hemphill et al. tested the ability of AAV1–AAV6 to transduce equine joint tissue explants (cartilage or synovium) and cultured chondrocytes or synoviocyte monolayers [149]. Tissue explants were considered as a model more closely related to in vivo transduction. AAV2 transduced around 80% of cells in cartilage explants, whereas AAV1 and AAV4 demonstrated near-zero transduction efficiency. Thus, AAV is a promising vector for RA gene therapy. 

One obstacle for the use of AAVs in RA gene therapy may come from adaptive immune responses. The prevalence of total IgG against different AAV serotypes among healthy humans is in the range of 37–72% [150]. Recently 34%, 83 out of 243 healthy blood donors, were found to be AAV-positive according to the PCR [151]. In most people, the immune response emerges 2 years after infection by wild-type AAV [143]. Interestingly, the longest reported transgene expression caused by a parenterally administered AAV is 10 years [152]. Thus, it is likely that the intended therapeutic effect may be exerted, even after the immune response to the vector. However, when using AAVs as vectors, the possible immune response should be considered, especially in the case of repeated administrations. Nonetheless, this circumstance may be resolved by repeated use of vectors constructed from different AAV serotypes [153]. Other methods are also possible. For example, synthetic variants obtained by structure-guided antigenic epitope evolution allows evading neutralizing antibodies (NAbs) [154]. Using an AAV capsid from other host species may also hide the virus from the pre-existing NAbs [155]. Empty capsids can act as bait for antibodies, promoting the distribution of the target vector [156]. Recently, it was demonstrated that synthetic vaccine particles encapsulating rapamycin coadministered with AAV vectors prevent the induction of anti-capsid humoral and cell-mediated responses [157]. Thus, immunogenicity does not seem to be an unpassable barrier to the use of AAV-based gene therapy. 

Many other attempts to create safe and efficient viral vectors have been made. Alphaviruses, flaviviruses, picornaviruses, rhabdoviruses, and paramyxoviruses are among the viruses that have been tried as delivery vehicles in viral-based gene therapy [158]. However, these approaches have not yet been applied to RA therapy, and most of them do not appear as the best choice in the case of RA. Viral vectors described in this manuscript are summarized in the Table 2.

## 4. Future Directions

Gene therapy is a method of treating or preventing a disease by interacting with the genetic apparatus of human somatic cells. This is achieved by transferring nucleic acids in the form of DNA or RNA into the patient’s body. It is noteworthy that these molecules do not necessarily transfer genetic information directly. Apart from well-described transient expression and genome editing tools, it may be, for example, a new class of “smart therapeutics” represented by nucleic acids with enzymatic activity. RNA-cleaving DNAzymes can cut mRNA, directly causing target gene silencing [161]. Additionally, potential drugs may regulate microRNA processing, thereby initiating target gene mRNA interference [162].

Mammalian articular cartilage is considered to have poor regenerative properties [163]. At the same time, other vertebrates can regenerate not only joints but also entire limbs [164]. Recently, it was demonstrated that human cartilage has a hitherto unappreciated innate regenerative capacity associated with microRNA expression [165]. MiRNA-21, miRNA-31, and miRNA-181c are upregulated during limb regeneration in zebrafish, bichir, and axolotl [164]. A significant gradient of miRNA expression (proximal to distal) was observed for miRNA-21 and miRNA-31 [165]. Moreover, miRNA-181c was enriched in osteoarthritis-affected cartilage as compared with healthy cartilage. Currently, it is unclear if these regeneration mechanisms can be used in RA treatment. 

RNA metabolism is another potential direction of RA gene therapy. The real transcriptomics universe may be much more complex than it was supposed to be. For example, modified ribonucleotides in siRNA evade immune response without loss of RNAi activity [166]. Recently, increased concentrations of adenosine deaminases acting on RNA (ADARs) were identified in the synovium of patients with RA [167]. Adenosine deamination results in the adenosine-to-inosine (A-to-I) editing event in mRNA and can modulate the level of protein expression. Cathepsin S is an extracellular matrix degradation enzyme involved in antigen presentation. The noncoding part of its mRNA demonstrated an increased number of A-to-I events in samples from patients with RA. Moreover, cathepsin S expression in the synovium was significantly increased in RA samples [167]. Interestingly, both ADAR expression and the individual adenosine RNA editing rate of cathepsin S decreased after treatment only in patients with a good clinical response to TNFis. Thus, the measurement of ADAR concentration may be a good marker for the prognosis of TNFi efficiency. As ADAR expression and activity are increased after stimulation with TNF-α, ADAR inhibitors may be considered a potential RA treatment.

## 5. Conclusions

In the context of the search for drugs against RA, the key problem is the unclear etiology of the disease. It is likely that the existing therapy is aimed at correcting the consequences of an unknown cause. At the same time, emerging data allows looking at the problem from a different perspective. Currently, there are dozens of potential RA gene therapy targets and comparing their significance is difficult. Risk/benefit and, potentially, cost/benefit ratios are not favorable for many classical gene therapy approaches, and the issue of complete cure at one shot or repeated administration of a transgene is important. Therefore, despite many trials and advancements, RA gene therapy remains in its infancy as compared to monogenic diseases.

## Figures and Tables

**Table 1:** Possible novel targets for rheumatoid arthritis (RA) therapy.

Type of Molecule	Possible Target	Effector	Model	Therapeutic Effect	References
Cytokine	IL-17	Human anti-IL-17 mAB (secukinumab)	Phase III clinical trials, patients with RA who have inadequate response to anti-TNF therapy	Significantly better clinical efficacy as compared with placebo, but the effect does not exceed that of treatment with abatacept.	[27,87]
Cytokine	IL-4, IL-10	IL4-10 FP	Mice, PGIA	Suppression of disease severity without inducing B cell hyperactivity. Suppression of articular cartilage damage in models for osteoarthritis.	[30]
MMP	MT1-MMP	MT1-MMP selective inhibitory antibody (DX-2400) and/or TNFR-Fc fusion protein	Mice, CIA	Reduction of cartilage degradation and disease progression. DX-2400 and TNFR-Fc acted synergistically.	[41]
MMP	MMP-9	mAB to MMP-9 (andecaliximab)	Phase Ib completed	Proved short-term safety; further studies are warranted.	[40]
miRNA	miR-203	Transfection with synthetic miR-203 precursor	Human, primary FLSs culture (RA and HC)	Overexpression in RA-FLS led to increased levels of MMP1, IL-6.	[53]
miRNA	miR-18a	Transfection with synthetic miR-18a precursor	Human, primary RA-FLSs culture	Overexpression in RA-FLS led to increased levels of MMP1, IL-6, and IL-8.	[54]
miRNA	miR-17	Transfection with synthetic miR-17 precursor	Human, primary RA-FLSs culture	Inhibited the TNF-α-induced IL-6, IL-8, MMP-1, and MMP-13 production in RA-FLSs.	[55]
miRNA	miR-19a/b	Transfection with miR-19a and miR-19b mimics	Human, primary RA-FLSs culture	Overexpression led to downregulation of IL-6 and MMP-3 secretion by controlling TLR2 expression.	[56]
miRNA	miR-20a	Transfection with miR-20 mimics	Human, primary RA-FLSs culture	Overexpression led to decreased IL-6 and CXCL10 release by RA-FLS.	[57]
miRNA	miR-135a	siRNA	Human, primary FLSs culture	Downregulation of miR-135a led to inhibited cell proliferation, migration, and invasion and promoted cell apoptosis through upregulation of PIK3R2 and inactivation of the PI3K/AKT signaling pathway.	[58]
miRNA	miR-21	siRNA (lentivirus)	Rats, CIA	Inhibition of miR-21 in RA-FLSs led to significant decrease in cell proliferation rates.	[59]
miRNA	miR-124a	Chemically synthetized miR-124a mimic	Human, primary RA-FLSs culture	Suppresses the proliferation and invasion of RA-FLSs	[60]
miRNA	miR-27a	Transfection with miR-27a	Human, primary RA-FLSs culture	Overexpression inhibited cell migration and invasion of RA-FLSs by targeting FSTL1 and restraining the TLR-4/NF-κB pathway.	[61]
lncRNA	GAPLINC	siRNA	Human, primary RA-FLSs culture	Decreased the migration and invasion of RA-FLSs as well as production of proinflammatory cytokines (IL-6 and IL-8) and MMPs.	[70]
lncRNA	NEAT1	Lentivirus-constructed short hairpin RNA interference, injection into joint	Mice, CIA	Inhibited differentiation of CD4+ T cells into Th17 cells through reducing level of STAT3 transcription factor.	[72]
lncRNA	UCA1	siRNA	Human, primary RA-FLSs culture	The downregulation of UCA1 expression increased the viability in normal FLSs, while overexpression of UCA1 in RA-FLSs inhibited the viability of cells.	[88]
lncRNA	LERFS	lncRNA Smart Silencer (RiboBio)	Human, primary FLSs culture (RA and HC)	Silencing of LERFS led to increased proliferation and migration of FLSs.	[71]
lncRNA	ZFAS1	shRNAs (lentivirus)	Human, primary FLSs culture (RA and HC)	Knockdown decreased MMP-2 and MMP-9 expression and thus suppressed migration and invasion of RA-FLSs through suppression of miR-27a.	[89]

**Table 2:** Viral vectors for RA gene therapy.

Type of Viral Vector	Unique Properties	Side Effects	Clinical Implication for RA Gene Therapy
Adenovirus	37-kb-large insert size for the gene of interest; adenoviruses are maintained in cells as an episome [159]	Potent immune response in the case of a systemically administered adenoviral vector	Adenoviral vectors are used primarily for applications in which an immune response is desirable. Autoimmune RA is not among the optimal applications for adenoviral delivery systems.
Retrovirus	9–12-kb-large insert size for the gene of interest; viral DNA is integrated into the host genome [160]	High risk of malignancies	The risk of insertional mutagenesis for retroviral vectors limits their clinical implication.
Lentivirus	9–12-kb-large insert size for the gene of interest; viral DNA is integrated into the host genome [160]	Risk of malignancies	Introduction of lentivirus-based constructs into clinical practice is limited and must include a very careful risk/benefit analysis, which is unlikely to be favorable in the case of RA.
AAV	4.8-kb-large insert size for the gene of interest [124];AAV genome mostly exists as an epichromosome	Good safety profile	AAV vectors are recognized as safe and effective, being one of the most promising methods of gene therapy.
